# Antiretroviral Therapy for Prevention of Tuberculosis in Adults with HIV: A Systematic Review and Meta-Analysis

**DOI:** 10.1371/journal.pmed.1001270

**Published:** 2012-07-24

**Authors:** Amitabh B. Suthar, Stephen D. Lawn, Julia del Amo, Haileyesus Getahun, Christopher Dye, Delphine Sculier, Timothy R. Sterling, Richard E. Chaisson, Brian G. Williams, Anthony D. Harries, Reuben M. Granich

**Affiliations:** 1 Department of HIV/AIDS, World Health Organization, Geneva, Switzerland; 2 Desmond Tutu HIV Centre, University of Cape Town, Cape Town, South Africa; 3 Department of Clinical Research, London School of Hygiene & Tropical Medicine, London, United Kingdom; 4 National Centre of Epidemiology, Instituto de Salud Carlos III, Madrid, Spain; 5 Stop TB Department, World Health Organization, Geneva, Switzerland; 6 HIV/AIDS, Tuberculosis, Malaria and Neglected Tropical Diseases Cluster, World Health Organization, Geneva, Switzerland; 7 Department of Medicine, Vanderbilt University School of Medicine, Nashville, Tennessee, United States of America; 8 Department of Medicine, Johns Hopkins University School of Medicine, Baltimore, Maryland, United States of America; 9 South African Centre for Epidemiological Modelling and Analysis, University of Stellenbosch, Stellenbosch, South Africa; 10 International Union Against Tuberculosis and Lung Disease, Paris, France; 11 Department of Infectious and Tropical Diseases, London School of Hygiene & Tropical Medicine, London, United Kingdom; MRC Clinical Trials Unit, United Kingdom

## Abstract

**Background:**

Human immunodeficiency virus (HIV) infection is the strongest risk factor for developing tuberculosis and has fuelled its resurgence, especially in sub-Saharan Africa. In 2010, there were an estimated 1.1 million incident cases of tuberculosis among the 34 million people living with HIV worldwide. Antiretroviral therapy has substantial potential to prevent HIV-associated tuberculosis. We conducted a systematic review of studies that analysed the impact of antiretroviral therapy on the incidence of tuberculosis in adults with HIV infection.

**Methods and Findings:**

PubMed, Embase, African Index Medicus, LILACS, and clinical trial registries were systematically searched. Randomised controlled trials, prospective cohort studies, and retrospective cohort studies were included if they compared tuberculosis incidence by antiretroviral therapy status in HIV-infected adults for a median of over 6 mo in developing countries. For the meta-analyses there were four categories based on CD4 counts at antiretroviral therapy initiation: (1) less than 200 cells/µl, (2) 200 to 350 cells/µl, (3) greater than 350 cells/µl, and (4) any CD4 count.

Eleven studies met the inclusion criteria. Antiretroviral therapy is strongly associated with a reduction in the incidence of tuberculosis in all baseline CD4 count categories: (1) less than 200 cells/µl (hazard ratio [HR] 0.16, 95% confidence interval [CI] 0.07 to 0.36), (2) 200 to 350 cells/µl (HR 0.34, 95% CI 0.19 to 0.60), (3) greater than 350 cells/µl (HR 0.43, 95% CI 0.30 to 0.63), and (4) any CD4 count (HR 0.35, 95% CI 0.28 to 0.44). There was no evidence of hazard ratio modification with respect to baseline CD4 count category (*p* = 0.20).

**Conclusions:**

Antiretroviral therapy is strongly associated with a reduction in the incidence of tuberculosis across all CD4 count strata. Earlier initiation of antiretroviral therapy may be a key component of global and national strategies to control the HIV-associated tuberculosis syndemic.

**Review Registration:**

International Prospective Register of Systematic Reviews CRD42011001209

*Please see later in the article for the Editors' Summary.*

## Abstract 2

In a systematic review and meta-analysis, Amitabh Suthar and colleagues investigate the association between antiretroviral therapy and the reduction in the incidence of tuberculosis in adults with HIV infection.

## Editors' Summary

**Background:**

Tuberculosis—a contagious bacterial infection— is a global public-health problem. In 2010, 8.8 million people developed active tuberculosis and 1.4 million people died from the disease. Tuberculosis can be cured by taking powerful antibiotics regularly for several months, and between 1995 and 2010, 46 million people with tuberculosis were successfully treated using DOTS—a directly observed antibiotic regimen designed by the World Health Organization (WHO). Now, though, the HIV epidemic is compromising global tuberculosis control efforts. HIV-positive people are very susceptible to tuberculosis because HIV, the virus that causes AIDS, destroys the immune system cells (including CD4 lymphocytes) that normally combat tuberculosis. In 2010, 1.1 million of the new (incident) cases of tuberculosis were among the 34 million people living with HIV, and 350,000 people died of HIV-associated tuberculosis, making tuberculosis the leading cause of death among HIV-positive people. To tackle HIV-associated tuberculosis, which occurs mainly in developing countries, WHO now recommends that HIV and tuberculosis programs use collaborative approaches such as the Three I's for HIV/TB strategy—intensified tuberculosis case-finding among HIV-positive people, isoniazid preventative therapy for HIV-positive people without active tuberculosis, and (tuberculosis) infection control in healthcare facilities, social settings, and households.

**Why Was This Study Done?:**

Despite progress in scaling up the Three I's for HIV/TB strategy, complementary interventions are still needed to prevent tuberculosis in HIV-positive people. Antiretroviral therapy (ART) lowers the viral load of people infected with HIV and restores their immune system function and could, therefore, prevent HIVassociated tuberculosis, in addition to treating HIV infection. WHO recommends ART for all HIV-positive adults with a CD4 count of less than 350 cells/μl of blood and for all HIVpositive, tuberculosis-positive individuals irrespective of their CD4 count. However, the evidence for ART's preventative impact on tuberculosis has not been systematically examined. Here, the researchers undertake a systematic review (a search that uses predefined criteria to identify all the research on a given topic) and a meta-analysis (a statistical method for combining the results of studies) to investigate the impact of ART initiated at various CD4 counts on the development of tuberculosis in HIV-positive adults in developing countries.

**What Did the Researchers Do and Find?:**

The researchers found 11 studies that compared tuberculosis incidence by ART status in HIV-infected adults over periods longer than six months on average in developing countries and undertook meta-analyses of these studies based on four categories of CD4 count at ART initiation (less than 200 cells/μl, 200–350 cells/μl, greater than 350 cells/μl, and any CD4 count). For all these categories, ART was strongly associated with a reduction in the incidence of tuberculosis. For example, the meta-analysis of the two studies that reported on participants in whom ART was initiated at a CD4 count less than 200 cells/μl yielded a hazard ratio (HR) of 0.16. That is, study participants starting ART when their CD4 count was below 200 cells/μl were about one-sixth as likely to develop tuberculosis as participants not receiving ART. In the metaanalysis of all 11 studies, study participants receiving ART were about one-third as likely to develop tuberculosis as study participants receiving no ART, irrespective of their CD4 count (HR 0.35). Importantly, the CD4 count at which ART was initiated did not significantly alter the magnitude of ART's preventive effect on tuberculosis development.

**What Do These Findings Mean?:**

These findings suggest that ART is strongly associated with a reduction in the incidence of tuberculosis in HIV-positive adults in developing countries, whatever the CD4 count at ART initiation. Because most of the studies in this meta-analysis were observational, these results do not show that ART causes a reduction in tuberculosis incidence—other unknown factors shared by the study participants who received ART may be responsible for their lower tuberculosis incidence. Moreover, factors such as variations in diagnostic methods among the studies included in this meta-analysis may have affected the accuracy of these findings. Nevertheless, the key finding that ART is associated with a significant reduction in tuberculosis cases among adults with CD4 counts greater than 350 cells//μl should be considered by healthcare providers, policymakers, and people living with HIV when weighing the benefits and risks of early ART initiation. It also suggests that early ART initiation (in combination with expanded HIV testing) could be a key component of future global and national strategies to control HIV-associated tuberculosis.

**Additional Information:**

Please access these websites via the online version of this summary at http://dx.doi.org/10.1371/journal.pmed.1001270.

- WHO provides information on all aspects of tuberculosis, including information on tuberculosis and HIV, on the Three I's for HIV/TB, and on ART for tuberculosis prevention (some information is in several languages)
- The TB/HIV Working Group is part of the Stop TB Partnership, which is working toward tuberculosis elimination; patient stories about tuberculosis/HIV co-infection are also available on their site
- The US Centers for Disease Control and Prevention has information about tuberculosis and about tuberculosis and HIV co-infection
- The US National Institute of Allergy and Infectious Diseases also has detailed information on all aspects of tuberculosis including HIV-associated tuberculosis
- Information is available from Avert, an international AIDS charity, on HIV-related tuberculosis (in English and Spanish), and from Aidsmap, a non-governmental organization, on HIV-associated tuberculosis

## Introduction

Tuberculosis and human immunodeficiency virus (HIV) are major threats to global public health. HIV infection is the strongest risk factor for tuberculosis and has fuelled its resurgence [1]. In 2010 there were an estimated 1.1 million incident cases of tuberculosis among the 34 million people living with HIV worldwide; 900,000 of these cases were among the 22.9 million Africans living with HIV [2],[3]. The 350,000 deaths among incident HIV-positive tuberculosis cases comprised 19% of all HIV-related deaths [2] and 24% of all tuberculosis deaths globally [3].

As part of the Millennium Development Goals, all 192 United Nations member states agreed to halt and decrease the annual mortality, incidence, and prevalence of tuberculosis and to increase the proportion of tuberculosis cases detected and cured under the DOTS strategy by 2015 [4]. The World Health Organization (WHO) and the Stop TB Partnership have endorsed the Millennium Development Goal targets and also aim to reduce the global annual incidence of active tuberculosis to less than one case per million population by 2050 [5]. While latest estimates indicate that the world is on track to achieve the Millennium Development Goal targets [3], achieving elimination will require a shift in strategy [6]–[8].

The DOTS strategy was largely developed in the pre-HIV era, and its implementation between 1995 and 2010 helped successfully treat 46 million people with tuberculosis and save 6.8 million lives [3]. While the DOTS strategy is essential for people with and without HIV, it is unlikely to reduce the incidence and prevalence of tuberculosis in countries where HIV is highly prevalent [9]. Given the importance of HIV as a driver of the tuberculosis epidemic in many regions, especially in Africa, where approximately 40% of incident tuberculosis cases in 2010 were associated with HIV [3], WHO recommends a range of collaborative activities through which HIV and tuberculosis programmes can address HIV-associated tuberculosis [10]. These include the Three I's for HIV/TB: intensified tuberculosis case-finding [11], isoniazid preventive therapy [11], and tuberculosis infection control [12]. Unfortunately only 178,144 people, a small fraction of the millions eligible, received isoniazid preventive therapy in 2010 [3]. The barriers contributing to this low coverage of isoniazid preventive therapy are complex and underscore the need for complementary interventions to prevent tuberculosis in adults with HIV [1],[11],[13].

In 2009, WHO recommended antiretroviral therapy for all adults with CD4 counts less than 350 cells/µl and for all tuberculosis patients irrespective of CD4 count [14]. In more recent years, accumulating evidence has pointed towards the potential of antiretroviral therapy scale-up to further contribute to control of the HIV-associated tuberculosis syndemic [15],[16],[17]. However, the evidence regarding antiretroviral therapy's preventive impact on tuberculosis has not undergone formal systematic review or synthesis. The objective of this study was to systematically review the effect of antiretroviral therapy on incident tuberculosis in developing countries across a range of CD4 cell count strata.

## Methods

### Conduct of Systematic Review

This systematic review was conducted in accordance with the PRISMA (Preferred Reporting Items for Systematic Review and Meta-Analyses) statement (Text S1) [18]. The investigators wrote a protocol and registered it with the International Prospective Register of Systematic Reviews (identification number: CRD42011001209) in March 2011 [19]. PubMed and Embase were systematically searched without language, publication, or date restrictions in August 2011, while African Index Medicus and LILACS (Latin American and Caribbean Health Science Literature Database) were systematically searched without language, publication, or date restrictions in February 2012.

### Search Strategy, Selection Criteria, and Data Extraction

The search strategies (Table S1) were designed with a librarian to identify studies reporting on the effect of antiretroviral therapy in preventing HIV-associated tuberculosis. Per recommendations from the PRISMA Group, eligibility criteria were based on key study characteristics: population, intervention, comparator, outcome, design, and length of follow-up [18]. Specifically, studies were included when (1) the study population was composed of adults (≥13 y) with HIV; (2) the intervention was antiretroviral therapy (defined as three or more antiretroviral drugs used in combination); (3) the comparator was no antiretroviral drugs; (4) the outcome was an incident case of tuberculosis; (5) the study design was a randomised trial, prospective cohort study, or retrospective cohort study; and (6) participants were followed for more than 6 mo (since viral suppression, immune recovery, and associated tuberculosis risk reduction is a time-dependent process [20]–[24] and tuberculosis rates during early antiretroviral therapy depend highly upon the intensity of screening for prevalent tuberculosis prior to antiretroviral therapy initiation [25]). The WHO International Clinical Trials Registry Platform, the Cochrane Central Register of Controlled Trials, the International Standard Randomised Controlled Trial Number Register, and ClinicalTrials.gov were searched for future and ongoing studies using the terms “antiretroviral” and “tuberculosis”. Experts in the field were also contacted to identify unpublished research or ongoing studies.

Tuberculosis transmission is complex and is influenced by biological, social, and economic factors [26]. Data from 134 countries indicate that development, measured by the Human Development Index, correlates with national tuberculosis incidence [27]. The Human Development Index is a composite national score of health (life expectancy at birth), education (expected years of schooling), and living standards (per capita gross national income) [28]. The Human Development Index categorises developed countries as those scoring in the top quartile and developing countries as those scoring below the top quartile [28]. Since developed countries collectively contributed less than 0.5% of all HIV-positive tuberculosis cases globally (Table S2), the scope of this systematic review was limited to developing countries to maximise the generalisability of the meta-analyses to countries facing the highest burden of HIV-associated tuberculosis.

**Figure 1:**
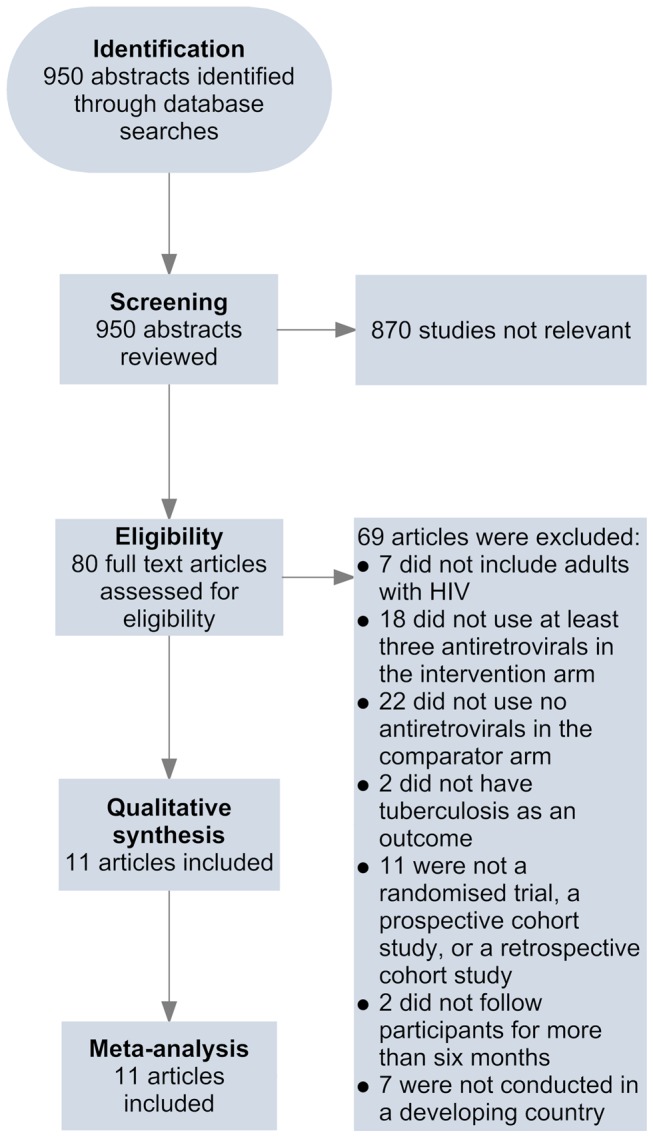
Flow of information through different phases of the review.

Two of the investigators, A. B. S. and D. S., independently screened abstracts of all retrieved articles from PubMed and Embase and then matched the full texts of all articles selected during screening against the inclusion criteria. A. B. S. and R. M. G. conducted this same process for the African Index Medicus and LILACS databases. Disagreements on which articles met the inclusion criteria were resolved by discussion. Articles meeting inclusion criteria were included in the review (Figure 1). A. B. S. and J. d. A. completed the data extraction using a standardised spreadsheet that collected information on the first author, year of publication, methods and design, study population, intervention and control, duration of follow-up, inclusion and exclusion criteria, outcomes, and losses to follow-up.

### Quality Assessment

Per recommendations from the Cochrane Collaboration [29], the Newcastle-Ottawa quality assessment scale was used to assess bias in studies included in this review [30]. This scale rates studies on three sources of bias based on eight criteria. Each criterion is worth one point except confounding, which is worth two points. Selection bias was assessed using four criteria: (1) representativeness of the cohort on antiretroviral therapy to the average adult on antiretroviral therapy in the community from which study participants were drawn, (2) representativeness of the cohort off antiretroviral therapy to the cohort on antiretroviral therapy, (3) ascertainment of antiretroviral therapy use, and (4) demonstration that prevalent tuberculosis was not present at the start of follow-up. To judge whether appropriate methods were used to address confounding, adjustment for baseline CD4 count was used for studies not reporting analyses in CD4 strata. Since a low body mass index is a key risk factor for developing tuberculosis in adults, irrespective of HIV status [8],[21],[31]–[34], adjustment for body mass index was used to judge whether appropriate methods were used to address confounding for analyses within CD4 strata. Measurement bias was assessed with three criteria: (1) microbiological (i.e., culture or acid-fast bacilli smear) confirmation of tuberculosis cases, (2) adequate follow-up to detect antiretroviral therapy's long-term preventive effect on tuberculosis (i.e., median follow-up of at least 1 y [20]–[24]), and (3) ≤30% of participants lost to follow-up during the study. Based on these criteria, studies were scored out of 100%. For this systematic review, studies scoring ≥67% were arbitrarily considered high methodological quality, those scoring 34%–66% were arbitrarily considered moderate methodological quality, and those ≤33% were arbitrarily considered low methodological quality.

Per recommendations from the Cochrane Collaboration [29], the Collaboration's Risk of Bias tool was used to assess bias in randomised trials meeting eligibility criteria. This tool rates studies on four sources of bias based on six criteria: (1) adequate sequence generation to gauge selection bias; (2) allocation concealment to gauge selection bias; (3) blinding of participants, personnel, and outcome assessors to gauge performance and detection bias; (4) incomplete outcome data to gauge attrition bias; (5) selective reporting to gauge reporting bias; and (6) a criterion for other forms of bias. Based on these criteria, trials were scored out of 100%.

### Statistical Analyses

Past WHO guidelines have used a CD4 threshold of 200 cells/µl [35] and 350 cells/µl [14] for initiation of antiretroviral therapy in asymptomatic adults. Given that there is considerable heterogeneity among different populations regarding CD4 counts directly after seroconversion and the subsequent rate of CD4 decline, the need for multiple strata above 350 cells/µl is population-specific [36]–[38]. Therefore, four categories based on CD4 at antiretroviral therapy initiation were used for the analytical component of this review: less than 200 cells/µl, 200 to 350 cells/µl, greater than 350 cells/µl, and any CD4 count. A funnel plot with the effect measures on the *x*-axis and standard error of the log for the effect measures on the *y*-axis was created to assess publication bias, and the Egger and Begg tests were used to test the funnel plot's symmetry. Since studies were similar enough to combine, meta-analyses were performed and statistical heterogeneity was assessed. Effect measures were entered as the natural log of the effect measure, and standard error as the natural log of (95% upper limit÷95% lower limit)÷3.92 [39]. Fixed-effects models assume that the magnitude and direction of an intervention's effect is identical across studies and that observed differences among study results are due solely to chance [29]. Random-effects models assume that the magnitude and direction of an intervention's effect is not identical across studies but follows a distribution [29]. Since it is possible that the magnitude and direction of antiretroviral therapy's preventive impact on tuberculosis could differ for reasons other than chance, random-effects models were used for all meta-analyses. χ and τ statistics require the number of events in each study arm to assess heterogeneity in the magnitude of effect across studies. Since these data were not available for all studies meeting inclusion criteria, *I* ^2^ statistics were used to measure heterogeneity [40]. *I* ^2^ values near 25% indicate low heterogeneity, values near 50% indicate moderate heterogeneity, and those above 75% indicate high heterogeneity [41]. The χ^2^ test, against the null hypothesis that there is no difference in the hazard ratio (HR) with respect to baseline CD4 count category, was used to test for hazard ratio modification. STATA version 10.0 was used for all analyses.

## Results

### Search Results

**Figure 2:**
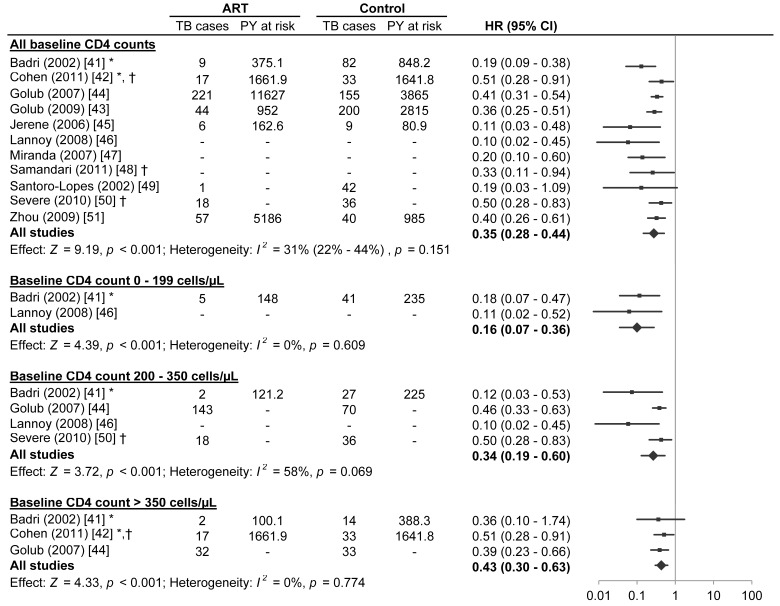
Antiretroviral therapy use and hazard of tuberculosis by baseline CD4 count. The centres of the squares represent study estimates, the centres of the quadrilaterals represent summary estimates, and the horizontal lines represent 95% confidence intervals. PY, person-years; –, data not reported; *, study effect measure is an incidence rate ratio; †, data are from a randomised controlled trial.

Eleven studies met the inclusion criteria for this systematic review (Tables 1 and 2) [42]–[52]. Four of these studies were from sub-Saharan Africa [42],[44],[46],[49], four were from South America [45],[47],[48],[50], one was from the Caribbean [51], one was from Asia [52], and one was from a combination of regions in sub-Saharan Africa, South America, and Asia [43]. Two studies reported effect estimates for baseline CD4 counts less than 200 cells/µl [42],[47], four studies reported effect estimates for baseline CD4 counts from 200 to 350 cells/µl [42],[45],[47],[51], and three studies reported effect estimates for baseline CD4 counts greater than 350 cells/µl [42],[43],[45] (Figure 2). One ongoing randomised study was identified in the Cochrane Central Register of Controlled Trials [53], three additional ongoing trials were identified in ClinicalTrials.gov [54]–[56], while no additional studies were found in the International Standard Randomised Controlled Trial Number Register or the WHO International Clinical Trials Registry Platform. Results on antiretroviral therapy's preventive impact on tuberculosis are not yet available from the ongoing trials [53]–[56].

**Table 1:** Characteristics of participants in studies meeting inclusion criteria.

Study (Year)	Country, Number (Percent) of Study Participants on ART/off ART	Inclusion and Exclusion Criteria	WHO Clinical Stage and CD4 Count at Baseline	Baseline BMI or Body Weight
Badri et al. [42] (2002)	South Africa, 264 (26%)/770 (74%)	Adults >15 y were included. Exclusion criteria: acute opportunistic infection, significant laboratory abnormalities, current evidence of active substance abuse, pregnancy or lactation, treatment with immune-modulating or systemic chemotherapeutic agents, or a diagnosis of tuberculosis that did not fulfil the case definition	46% and 29% of those on and off ART, respectively, were stage 3 or 4. Median CD4 254 (IQR 140 to 364) cells/µl in those on ART and median CD4 303 (IQR 159 to 468) cells/µl in those off ART	Not reported
Cohen et al. [43] (2011)	Botswana, Brazil, India, Kenya, Malawi, South Africa, Thailand, and Zimbabwe, 886 (50%)/877 (50%)	Adults ≥18 y with CD4 counts from 350 to 550 cells/µl were included. Adults with a current or previous AIDS-defining illness or previous exposure to any ART drugs (except for mothers exposed during pregnancy) were excluded	Staging distribution not reported. Median CD4 442 (IQR 373 to 522) cells/µl in those starting ART at 350–550 cells/µl. Median CD4 428 (IQR 357 to 522) cells/µl in those deferring ART initiation to below 350 cells/µl	Not reported
Golub et al. [45] (2007)	Brazil, 8,129 (74%)/2,898 (26%)	Adults who had made ≥1 visits to their primary care clinic were included. Adults who attended the clinic to collect ART prescribed by a private physician, who died before the end of follow-up, or for whom dates were not available were excluded	Staging distribution not reported. 22.5% with CD4<200 cells/µl, 24.9% with CD4 200–349 cells/µl, 22.5% with CD4 350–499 cells/µl, and 30% with CD4≥500 cells/µl	Not reported
Golub et al. [44] (2009)	South Africa, 820 (30%)/1,958 (70%)	Adults >18 y were included. Those without CD4 results were excluded	Staging distribution not reported. Median CD4 266 (IQR 139 to 439) cells/µl	Median BMI 23.6 (IQR 20.4 to 28.1) kg/m^2^
Jerene et al. [46] (2006)	Ethiopia, 180 (49%)/185 (51%)	Adults ≥15 y with symptomatic HIV disease (WHO stage 2 to 4) were included	12% were stage 2, 69% were stage 3, and 19% were stage 4. CD4 distribution not available	Not reported
Lannoy et al. [47] (2008)	Brazil, 134 (53%)/118 (47%)	Adults were excluded if they lacked clinical records, received healthcare at private hospitals, or died within the first month after HIV diagnosis	Staging distribution not reported. Median CD4 196 (IQR 59 to 418) cells/µl	Not reported
Miranda et al. [48] (2007)	Brazil, 306 (80%)/76 (20%)	Adults were excluded if they attended the clinic only once or were <18 y of age, pregnant, or wards of the state	12% were stage 1 or 2, 82% were stage 3 or 4, and 6% were unstaged. 34% with CD4<350 cells/µl and 66% with CD4≥350 cells/µl	Not reported
Samandari et al. [49] (2011)	Botswana, 946 (47%)/1,049 (53%)	Adults ≥18 y without cough, weight loss, night sweats, other acute illnesses, previous isoniazid preventive therapy, tuberculosis treatment within the previous 3 y, neutrophil count <1.0×10^9^/l, or an abnormal chest radiograph were included	Staging distribution not reported. Median CD4 297 (IQR 172 to 449) cells/µl	336 (17%) underweight, 1,056 (53%) normal, 328 (16%) overweight, and 174 (9%) obese
Santoro-Lopes et al. [50] (2002)	Brazil, 41 (17%)/195 (83%)	Adults with ≥1 CD4 percentage <15% were included	Staging distribution not reported. 23% had CD4 percentage ≤7% and 77% had CD4 percentage ≥7%	Not reported
Severe et al. [51] (2010)	Haiti, 380 (49%)/393 (51%)	Adults ≥18 y with a baseline CD4 count from 200 to 350 cells/µl within 45 d of enrolment were eligible. Adults with a history of a WHO stage 4 event or who had received ART in the past were excluded	Among those starting ART at 200–350 cells/µl: 33% were stage 1, 49% were stage 2, 18% were stage 3. Among those starting ART at <200 cells/µl: 31% were stage 1, 54% were stage 2, and 63% were stage 3. Median CD4 280 (IQR 250 to 305) and 282 (IQR 250 to 310) cells/µl in those starting ART at 200–350 and <200 cells/µl, respectively	Median BMI 21.3 (IQR 19.6 to 23.7) and 21.0 (IQR 9.2 to 23.4) kg/m^2^ in those starting ART at 200–350 and <200 cells/µl, respectively
Zhou et al. [52] (2009)	17 sites in the Asia-Pacific region, 2,449 (75%)/830 (25%)	Adults with at least one prospective follow-up visit were included	53% with CDC stage A, 19% with CDC stage B, and 31% with CDC stage C. 32% with CD4<200 cells/µl, 19% 201300 cells/µl, and 49%>300 cells/µl	Not reported

ART, antiretroviral therapy; BMI, body mass index; CDC, US Centers for Disease Control and Prevention; IQR, interquartile range.

**Table 2:** Methods of studies meeting inclusion criteria.

Study (Year)	Study Design/Dates	Duration of Follow-Up (Months)	Baseline TB Screening and Exclusion	Definition of ART	Definition of TB	Analytical Method/Variables Used	IPT and How It Was Addressed	Losses to Follow-Up and How They Were Addressed
Badri et al. [42] (2002)	PCS/1992–2001	Mean 16.8 (SD 8.3) and 13.2 (SD 15.5) for those on and off ART, respectively	Screening not reported. Participants with TB at baseline were excluded	2 NRTIs+NNRTI, PI, or third NRTI	Definite TB was culture- or autopsy-confirmed. Probable TB was the presence of AFB or histological finding of caseating granulomata	Poisson regression/baseline CD4, WHO clinical stage, and socioeconomic status	Participants who received IPT in the 6 mo prior to baseline were excluded	Not reported
Cohen et al. [43] (2011)	RCT/2007–2011	Median 20.4	Screening not reported. Participants with TB at baseline were excluded	≥3 antiretrovirals	AIDS Clinical Trials Group definition as confirmed or probable [97]	IRR/none	IPT was available according to local guidelines at study sites	4 of the 3,538 participants (0.12%) were not able to be contacted. Analytical methods not reported
Golub et al. [45] (2007)	RCS/2003–2005	24	TST. Episodes of TB diagnosed within 4 wk of enrolment were excluded	≥3 antiretrovirals (per national guidelines)	Signs and symptoms compatible with TB on the basis of chest radiographs, sputum AFB smears, and response to anti-TB therapy	Cox proportional hazards/baseline age, sex, IPT history, TB history, CD4, HIV viral load, and TST	Participants on IPT allocated person-time in other study arms	Not reported
Golub et al. [44] (2009)	PCS/2003–2007	Median 22.8 in those on ART and median 12 in those off ART	Screening not reported. Adults with a history of TB or who developed TB ≤60 d of baseline were excluded	≥3 antiretrovirals (per national guidelines	TB diagnoses were based on microbiological confirmation, clinical diagnoses, and reports of being started on anti-TB therapy	Cox proportional hazards/baseline CD4, gender, clinic location, and age	Participants on IPT allocated person-time in other study arms	Not reported
Jerene et al. [46] (2006)	PCS/2003–2005	Median 12.5 (IQR 5.25 to 17) in those on ART and median 4.75 (IQR 2.5 to 8.5) in those off ART	Not reported	≥3 antiretrovirals	AFB sputum examinations, radiographic abnormalities, initiation of anti-TB therapy, and clinical suspicion were used to diagnose TB	Cox proportional hazards/oral thrush, diarrhoea, total lymphocyte count, anaemia, and BMI	Not reported	76% and 64.9% of the participants on and off ART, respectively, were under follow-up at the end of the study. Person-time lost to follow-up was censored
Lannoy et al. [47] (2008)	RCS/1998–2003	60	Not reported	Antiretrovirals for ≥3 mo starting from the cohort inception date	TB was identified using cultures, AFB smears, histological findings, or compatible clinical features (TB confirmed by having a good response to anti-TB therapy)	Cox proportional hazards/baseline CD4 ≤200 cells/µl	Not reported	Losses not reported. Patients who did not complete the follow-up period and remained TB-free were censored at the last medical evaluation available before death
Miranda et al. [48] (2007)	RCS/1995–2001	Mean 37.5 until the last clinic visit	Screening not reported. Participants who developed TB within 30 d of the first clinic visit were excluded	2 NRTIs+PI, 2 NRTIs+NNRTI, or NRTI+NNRTI+PI	Confirmed TB was culture-confirmed, probable TB was AFB positive, and presumptive TB was based on an abnormal chest X-ray, caseous granulomatous reaction, or the prescription of anti-TB treatment	Cox proportional hazards/baseline CD4, TST result, use of IPT, and history of hospitalisation, incarceration, intravenous drug use, and TB	IPT was included in the final model	Not reported
Samandari et al. [49] (2011)	RCT/2004–2009	36	Participants with weight loss, cough, night sweats, or past TB treatment were excluded	2 NRTIs+NNRTI	Clinical presentation consistent with TB and response to anti-TB therapy	Cox proportional hazards/baseline CD4, TST result, use of IPT	Provided regardless of TST status, included in the final model	11 participants (0.55%) were lost to follow-up and excluded from the analyses
Santoro-Lopes et al. [50] (2002)	PCS/1991–1998	Median 22 (range 12.9 to 39.5)	Participants with previous TB were excluded	2 NRTIs+PI	Culture confirmation, clinical symptoms, favourable response to anti-TB therapy, presence of AFB in sputum, or radiological findings were used to diagnose TB	Cox proportional hazards/none	Follow-up accrued after participants started IPT was censored	4 of 41 (10%) and 47 of 214 (22%) patients on ART and off ART, respectively, were lost to follow-up. Analytical methods not reported
Severe et al. [51] (2010)	RCT/2005–2009	Median 21	Symptoms suggestive of TB used for screening. 43 participants with TB at enrolment were excluded	2 NRTIs+NNRTI/PI	American Thoracic Society case definition [98]	Cox proportional hazards/none	Provided to those with a positive TST skin test	19 and 18 participants randomised to start ART at 200–350 and <200 cells/µl, respectively, were lost to follow-up. Analytical methods not reported
Zhou et al. [52] (2009)	PCS/2003–2007	More than 12 for participants on ART, not reported for participants off ART	Screening not reported. TB cases that developed within 7 d of cohort entry were considered prevalent and excluded from incident analyses	Undefined, although 73% of those on ART were on 2 NRTIs+NNRTI	Definitive cases were culture-confirmed. Presumptive cases demonstrated AFB in a clinical/histopathological specimen, signs or symptoms compatible with TB, or resolved disease upon initiation of anti-TB therapy	Cox proportional hazards/age, HIV transmission route, CDC clinical class, baseline CD4, TB history, and country where care was received	13 of the 17 sites did not offer IPT. Four provided it to participants with CD4<200, positive TSTs, or a recent TB patient contact. Not included in model	Losses not reported. Follow-up was censored after the date of the most recent visit

AFB, acid-fast bacilli; ART, antiretroviral therapy; BMI, body mass index; CDC, US Centers for Disease Control and Prevention; IPT, isoniazid preventive therapy; IQR, interquartile range; NNRTI, non-nucleoside reverse transcriptase inhibitor; NRTI, nucleoside reverse transcriptase inhibitor; PCS, prospective cohort study; PI, protease inhibitor; RCS, retrospective cohort study; RCT, randomised controlled trial; SD, standard deviation; TB, tuberculosis; TST, tuberculin skin test.

### Quality Assessment

The assessment of bias indicated that four studies were of high methodological quality [43],[48],[49],[51], five studies were of moderate methodological quality [42],[44],[45],[47],[50], and two studies were of low methodological quality [46],[52] (Table 3). There appeared to be limited bias in the three randomised controlled trials identified [43],[49],[51] (Table 4).

**Table 3:** Newcastle-Ottawa quality assessment scale for studies meeting inclusion criteria.

Study (Year)	Selection Bias				Confounding		Measurement Bias			Study Score
	Representativeness of the Cohort on ART to the Average Adult on ART from the Community	Representativeness of the Cohort off ART to the Cohort on ART	Ascertainment of ART Use	Demonstration That Prevalent Tuberculosis Was Not Present at the Start of Follow-Up	For Estimates Regardless of Baseline CD4, Model Adjusted for CD4	For Estimates Reported in CD4 Strata, Model Adjusted for BMI	All Cases Microbiologically Confirmed	Median or Mean Follow-Up of at Least 1 y	≤30% of Participants Were Lost to Follow-Up during the Study	
Badri et al. [42] (2002)	1	0	1	1	2	0	0	1	0	55%
Cohen et al. [43] (2011)	1	1	1	1	NA	NA	0	1	1	86%
Golub et al. [45] (2007)	1	1	0	0	2	0	0	1	0	45%
Golub et al. [44] (2009)	1	1	0	0	2	NA	0	1	0	56%
Jerene et al. [46] (2006)	1	0	1	0	0	NA	0	0	1	33%
Lannoy et al. [47] (2008)	1	1	0	0	2	0	0	1	0	45%
Miranda et al. [48] (2007)	1	1	1	0	2	NA	0	1	0	67%
Samandari et al. [49] (2011)	1	1	1	1	NA	NA	1	1	1	100%
Santoro-Lopes et al. [50] (2002)	1	1	1	0	0	NA	0	1	1	56%
Severe et al. [51] (2010)	1	1	1	1	NA	NA	1	1	1	100%
Zhou et al. [52] (2009)	1	1	0	0	0	NA	0	0	0	22%

A score of 0 indicates “no”; a score of 1 or 2 indicates “yes”. Studies scoring ≥67% were considered high methodological quality, 34%–66% were considered moderate methodological quality, and ≤33% were considered low methodological quality. Given that the distribution of possible confounders in randomised controlled trials is related to chance alone, randomised controlled trials were not assessed for confounding.

ART, antiretroviral therapy; BMI, body mass index; NA, not applicable.

**Table 4:** Bias assessment for randomised controlled trials meeting inclusion criteria.

Study (Year)	Adequate Sequence Generation	Allocation Concealment	Blinding of Participants, Personnel, and Outcome Assessors	Incomplete Outcome Data Addressed	Free of Selective Reporting	Free of Other Bias	Study Score
Cohen et al. [43] (2011)	1	1	0	1	1	1	83%
Samandari et al. [49] (2011)	1	1	1	1	1	1	100%
Severe et al. [51] (2010)	1	1	0	1	1	1	83%

A score of 0 indicates “no”; a score of 1 indicates “yes”.

### Meta-Analyses

A meta-analysis of all eleven studies meeting inclusion criteria found that antiretroviral therapy is strongly associated with a reduction in tuberculosis incidence across all baseline CD4 counts (HR 0.35, 95% confidence interval [CI] 0.28 to 0.44; *p*-value for effect <0.001; *p*-value for heterogeneity = 0.151). Inspection of the funnel plot (Figure S1) suggested possible publication bias (Begg test *p* = 0.12; Egger test *p* = 0.02).

Two studies reported on participants with baseline CD4 counts less than 200 cells/µl. Badri et al. [42] (adjusted incidence rate ratio [IRR] 0.18, 95% CI 0.07 to 0.47) and Lannoy et al. [47] (IRR 0.11, 95% CI 0.02 to 0.52; Text S2) reported that antiretroviral therapy was associated with a reduction in tuberculosis incidence. A meta-analysis of these two studies found that antiretroviral therapy is strongly associated with a reduction in tuberculosis incidence in adults with baseline CD4 counts less than 200 cells/µl (HR 0.16, 95% CI 0.07 to 0.36; *p*-value for effect <0.001; *p*-value for heterogeneity = 0.609).

Four studies reported on participants with baseline CD4 counts from 200 to 350 cells/µl. Badri et al. [42] (adjusted IRR 0.12, 95% CI 0.03 to 0.53), Lannoy et al. [47] (adjusted HR 0.10, 95% CI 0.02 to 0.45), Golub et al. [45] (adjusted HR 0.46, 95% CI 0.33 to 0.63), and Severe et al. [51] (HR 0.50, 95% CI 0.29 to 0.83) reported that antiretroviral therapy was associated with a reduction in tuberculosis incidence. The meta-analysis of these four studies found that antiretroviral therapy is strongly associated with a reduction in tuberculosis incidence in adults with baseline CD4 counts from 200 to 350 cells/µl (HR 0.34, 95% CI 0.19 to 0.60; *p*-value for effect <0.001; *p*-value for heterogeneity = 0.069).

Three studies reported on participants with baseline CD4 counts above 350 cells/µl. Cohen et al. [43] (IRR 0.51, 95% CI 0.28 to 0.91; Text S2), Badri et al. [42] (adjusted IRR 0.36, 95% CI 0.10 to 1.74), and Golub et al. [45] (adjusted HR 0.39, 95% CI 0.23 to 0.66) reported that antiretroviral therapy was associated with a reduction in tuberculosis incidence, although Badri's estimate lacked statistical significance. The meta-analysis of these three studies indicated that antiretroviral therapy is strongly associated with a reduction in tuberculosis incidence in adults with CD4 counts above 350 cells/µl (HR 0.43, 95% CI 0.30 to 0.63; *p*-value for effect <0.001; *p*-value for heterogeneity = 0.774).

**Figure 3:**
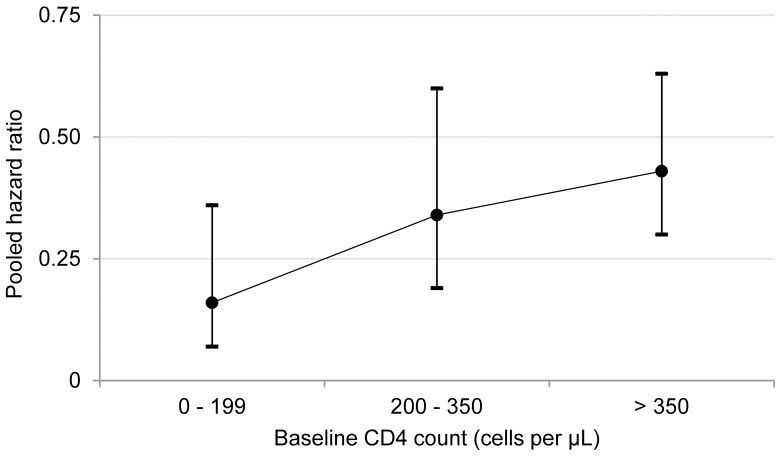
Antiretroviral therapy use and pooled hazard ratios of tuberculosis by baseline CD4 count. The circles represent pooled estimates, and the vertical lines represent 95% confidence intervals. The *p*-value for hazard ratio modification by baseline CD4 count category is 0.20. *I* ^2^ values for the 0–200, 201–350, and greater than 350 cells/µl categories are 0%, 58%, and 0%, respectively.

Visual inspection of the hazard ratios and confidence intervals for the three CD4 categories suggested a possible gradient in antiretroviral therapy's effect in relation to baseline CD4 count (Figure 3); however, there was no evidence of hazard ratio modification with respect to baseline CD4 count category using the χ^2^ test (*p* = 0.20).

## Discussion

This systematic review indicates that antiretroviral therapy is strongly associated with a reduction in tuberculosis incidence in adults with CD4 counts (1) less than 200 cells/µl (HR 0.16, 95% CI 0.07 to 0.36), (2) from 200 to 350 cells/µl (HR 0.34, 95% CI 0.19 to 0.60), (3) greater than 350 cells/µl (HR 0.43, 95% CI 0.30 to 0.63), and (4) at any level (HR 0.35, 95% CI 0.28 to 0.45). This study was a rigorous systematic literature review that focused exclusively on studies from developing countries and included very recent studies that provided data on adults with high baseline CD4 cell counts. These factors enabled what is, to our knowledge, the first ever estimate of antiretroviral therapy impact stratified by baseline CD4 category. The finding that antiretroviral therapy is strongly associated with a reduction in tuberculosis incidence across all CD4 counts is consistent with an earlier meta-analysis that included studies from developed and developing countries [13]. That meta-analysis, the meta-analyses reported here, and a previous comparative analysis of data from developed and developing countries [57] support the conclusion that tuberculosis risk reduction is similar regardless of country.

Nine of the 11 studies meeting the inclusion criteria were of moderate or high methodological quality (Tables 3 and 4). However, there were some methodological limitations that need to be considered when evaluating the strong association between antiretroviral therapy and the reduction of tuberculosis incidence. Since diagnostic capabilities differed by country and study site, some studies did not microbiologically confirm tuberculosis cases, which could induce measurement bias. Moreover, one of the studies found that earlier antiretroviral therapy was associated with a decrease only in extrapulmonary tuberculosis, while the others did not make a distinction between pulmonary and extrapulmonary disease [43]. Stratifying by disease site in future studies may be useful in explaining the contribution of antiretroviral therapy in preventing different types of tuberculosis. Since tuberculosis incidence rates during early antiretroviral therapy depend highly upon the efficiency of tuberculosis screening prior to antiretroviral therapy initiation [25], prevalent cases of tuberculosis are often unmasked soon after antiretroviral therapy initiation [20],[58]. Despite efforts to screen for prevalent tuberculosis at study baseline, it is difficult for investigators to establish definitively whether tuberculosis cases that develop soon after antiretroviral therapy initiation are truly incident cases. This uncertainly could introduce measurement bias into studies with a short period of follow-up. Furthermore, the complexity and expense of conducting randomised controlled trials means that most of our data were derived from observational studies. Although our analyses included data from randomised controlled trials, the potential for unmeasured confounding in prospective and retrospective cohort studies makes attempts to reliably establish causal effect more difficult. For example, in some studies there is potential for unmeasured confounding due to isoniazid preventive therapy (Table 2). Nonetheless, our finding that there is no hazard ratio modification with respect to baseline CD4 count is consistent with the randomised controlled trials, in which the reduction in tuberculosis incidence when initiating antiretroviral therapy at 200 to 350 cells/µl (HR 0.50, 95% CI 0.28 to 0.83) [51] was nearly identical to the reduction in tuberculosis incidence when initiating antiretroviral therapy above 350 cells/µl (IRR 0.51, 95% CI 0.28 to 0.91) [43]. These randomised controlled trial stratum estimates were also very similar to the 63% and 57% reductions obtained in the meta-analyses for the categories 200–350 cells/µl and greater than 350 cells/µl, respectively.

The meta-analyses may have limitations in the statistical methodology used. Since laboratory capabilities differed by country and study site, some of the studies did not adjust for baseline CD4 count, body mass index, smoking, and/or diabetes, which could confound results. Both incidence rate ratios and hazard ratios calculate events over person-time at risk; however, they rely on different methodologies depending on the nature of the data that are collected [59]. Given similarities in study methods (Table 2), the meta-analyses in this systematic review combined hazard ratios and incidence rate ratios from randomised controlled trials and cohort studies. A meta-regression of all studies included in the meta-analysis for all CD4 counts found that the type of effect measure (i.e., hazard ratio or incidence rate ratio) did not explain the heterogeneity in the magnitude of effect (*p* = 0.80). Although the χ^2^ test suggested no hazard ratio modification, inclusion of more strata and additional study estimates could improve this assessment. Since some studies contributed tuberculosis cases to CD4-stratum estimates and to estimates across all CD4 counts, the data used for the meta-analyses are not independent. Although there was mixed evidence of publication bias in this systematic review, the power to detect publication bias increases as the number of studies included in meta-analyses increases, and additional studies could strengthen the assessment of publication bias for antiretroviral therapy's preventive impact on tuberculosis [60]. While heterogeneity for the meta-analysis including all CD4 counts was calculated using *I* ^2^ statistics and a 95% confidence interval, calculating *I* ^2^ 95% confidence intervals for CD4 categories was not possible because of the limited number of studies within CD4 strata. Although the meta-analyses included antiretroviral therapy status and baseline CD4 count, other analyses exploring community tuberculosis incidence, community tuberculosis prevalence, participant history of tuberculosis, CD4 cell count recovery, and viral suppression might have provided additional insight into antiretroviral therapy's preventive impact on tuberculosis if these variables had been collected systematically in all studies. Finally, the validity of meta-analyses is subject to proper analyses by investigators in included studies. Two of the studies' 95% confidence intervals [46],[48] have asymmetry on the logarithmic scale. These two studies were included in the meta-analysis for all CD4 counts. In order to determine whether these studies introduced bias into our results, we ran a sensitivity analysis without them and found the results to be nearly identical (HR 0.35, 95% CI 0.28 to 0.44, with all studies, versus HR 0.38, 95% CI 0.31 to 0.46, without [46] and [48]).

While there are many potential benefits to providing earlier antiretroviral therapy, one risk of providing antiretroviral therapy to people with CD4 counts above 350 cells/µl is that it may compromise high adherence rates and potentially lead to widespread antiretroviral resistance. While this is plausible, a randomised trial has shown that adherence counselling facilitated greater than 95% adherence to antiretroviral therapy in 79% of participants initiating antiretroviral therapy above 350 cells/µl and 74% of participants initiating antiretroviral therapy below 350 cells/µl [43]. Additionally, observational data indicate that the risk of drug resistance is higher among people who started antiretroviral therapy below 350 cells/µl relative to those who started antiretroviral therapy above 350 cells/µl [61]. There is also concern that the risk of life-threatening antiretroviral therapy toxicity could be higher among people with CD4 counts above 350 cells/µl; however, a randomised trial indicates that the risk of life-threatening adverse events is similar in those initiating antiretroviral therapy above 350 cells/µl and those initiating antiretroviral therapy below 350 cells/µl (14% of participants in each study arm experienced such an event, *p* = 0.64) [43]. Results from surveillance and future trials [54]–[56] are awaited to confirm or refute these adherence and toxicity findings. Meanwhile, it is important to continue to scale up antiretroviral therapy to achieve universal access goals while also carefully conducting national surveillance of antiretroviral toxicity [62] and antiretroviral resistance [63].

While our analyses clearly show that antiretroviral therapy is strongly associated with a reduction in tuberculosis incidence in adults with HIV, its role in long-term tuberculosis elimination is more complex [6]–[8],[15]. Antiretroviral therapy's effect on the population incidence of tuberculosis depends on HIV prevalence and the extent to which antiretroviral therapy (1) reduces HIV transmission, (2) increases patient life expectancy, (3) reduces the annual risk of tuberculosis, and (4) reduces subsequent tuberculosis transmission. Dynamic models have suggested that antiretroviral therapy reduces new HIV infections and that increasing antiretroviral therapy coverage in people living with HIV will lower the population tuberculosis incidence [15]. Indeed, programmatic data thus far indicate that antiretroviral therapy scale-up is associated with reductions in tuberculosis incidence of 33% and 24% in high-burden Malawian and South African communities [64],[65]. Earlier antiretroviral therapy initiation could lead to a more substantial reduction in population tuberculosis incidence [15]. Expansion of antiretroviral therapy may also reduce HIV incidence at the city [66],[67], district [68],[69], and national levels [70],[71], while decreasing tuberculosis mortality [72]–[74] and HIV-related mortality [75]–[78].

Operationally, antiretroviral therapy's impact on tuberculosis control depends on (1) changes that facilitate access to HIV testing and linkage to care earlier in the course of HIV infection, (2) when national guidelines and programme implementation allow people to initiate antiretroviral therapy, (3) sustaining high adherence to antiretroviral therapy, and (4) improving long-term retention rates [16],[17],[79]. WHO recommends provider-initiated HIV testing and counselling in all health facilities in generalised (i.e., antenatal HIV prevalence ≥1%) epidemics [80]. Unfortunately, Demographic and Health Surveys indicate that only approximately 11% of people aged 15–49 y in generalised epidemics reported receiving an HIV test in the previous year [81], and that many people with HIV enrol onto antiretroviral therapy many years after HIV seroconversion, after the development of tuberculosis and other life-threatening illnesses, and after transmitting HIV to others [82]. A cluster-randomised trial recently found that community-based HIV testing detects approximately four times as many people with HIV as health-facility-based testing alone [83], and a 1-wk community-based multi-disease campaign recently tested 47,311 Kenyans (87% of the target sexually active population 15–49 y of age) and found that HIV-positive participants tested positive earlier in the course of their HIV infection (median 541 cells/µl in the campaign, [84]) than patients identified via health-facility-based approaches [82]. In order to harness the lifespan, HIV transmission, and tuberculosis prevention benefits of antiretroviral therapy, HIV programmes in countries with a high HIV prevalence need to expand HIV testing coverage and could consider offering community-based HIV testing, with linkage to antiretroviral therapy for those eligible, regularly to the general public [85].

WHO's Policy on HIV/TB Collaborative Activities currently recommends the Three I's for HIV/TB: intensified tuberculosis case-finding [11], isoniazid preventive therapy [11], and infection control [12] to prevent tuberculosis in people with HIV. WHO infection control guidelines recommend administrative, managerial, engineering, and personal respiratory methods to avoid nosocomial tuberculosis transmission, such as logistical changes to avoid patient congestion, and early identification and diagnosis of tuberculosis patients in healthcare facilities, congregate settings, and households [12]. Intensified tuberculosis case-finding involves screening people with HIV for current cough, night sweats, fever, and weight loss at every clinical encounter [11]. Those without any of these symptoms have a very low probability of having tuberculosis (98% negative predictive value in settings with a tuberculosis prevalence of 5% [86]) and should be initiated on isoniazid preventive therapy [11].

Isoniazid stops *Mycobacterium tuberculosis* replication during latent infection and reduces tuberculosis incidence by 33% [87]. WHO has recommended isoniazid preventive therapy for prevention of tuberculosis in adults with HIV since 1993 [11],[88],[89]; however, only a small fraction of the millions eligible received isoniazid preventive therapy in 2010 [3]. Antiretroviral therapy causes viral suppression and immune recovery, which reduces tuberculosis incidence by 65% across all CD4 counts. Initiating antiretroviral therapy as early as possible strengthens the WHO Three I's for HIV/TB strategy by building upon antiretroviral therapy's synergy with isoniazid preventive therapy. Indeed, observational studies from South Africa [44],[90], Brazil [45], and 16 other countries [91] indicate that combined isoniazid preventive therapy and antiretroviral therapy was superior to antiretroviral therapy or isoniazid preventive therapy alone in reducing tuberculosis incidence among adults with HIV. This finding was recently confirmed through a cluster-randomised trial in Brazil, where isoniazid preventive therapy reduced tuberculosis incidence among Brazilians who remained in care and received antiretroviral therapy [92]. These data suggest that antiretroviral therapy and isoniazid preventive therapy work by complementary mechanisms and that simultaneous use substantially decreases tuberculosis incidence in adults with HIV. Results from other ongoing trials assessing the synergy between antiretroviral therapy and isoniazid preventive therapy are eagerly awaited [54],[56], and ecological, operational, and clinical research on the impact of scaling up antiretroviral therapy and the Three I's for HIV/TB on community and/or national tuberculosis incidence rates is needed [93].

In conclusion, antiretroviral therapy is a potentially safe, well-tolerated, and HIV-transmission-interrupting intervention [43],[94] necessary to increase life expectancy in people with HIV [75]–[78]. There has been considerable debate on the optimal timing to start antiretroviral therapy in asymptomatic adults with HIV. Published results from ongoing randomised trials are expected in 2016 and are eagerly awaited [54],[55]. This review found that antiretroviral therapy is strongly associated with a reduction in tuberculosis incidence in adults with HIV across all CD4 cell counts. Our key finding that antiretroviral therapy has a significant impact on preventing tuberculosis in adults with CD4 counts above 350 cells/µl is consistent with studies from developed countries [95],[96] and will need to be considered by healthcare providers, researchers, policymakers, and people living with HIV when weighing the benefits and risks of initiating antiretroviral therapy above 350 cells/µl.

## Supporting Information

Figure S1 Funnel plot for studies meeting inclusion criteria, providing an estimate for all CD4 counts.
(PDF)

Table S1 Search strategies for the PubMed, Embase, LILACS, and African Index Medicus databases.
(PDF)

Table S2 HIV-associated tuberculosis cases in developed countries [3],[28].
(PDF)

Text S1 PRISMA checklist.
(PDF)

Text S2 Calculation of incidence rate ratios and 95% confidence intervals.
(PDF)

## Abbreviations

CI: confidence interval
HIV: human immunodeficiency virus
HR: hazard ratio
IRR: incidence rate ratio
WHO: World Health Organization
