# Supplementary material for: Antiretroviral Therapy for Prevention of Tuberculosis in Adults with HIV: A Systematic Review and Meta-Analysis
Source: PLoS Med. 2012 Jul 24;9(7):e1001270. doi: 10.1371/journal.pmed.1001270 (PMC3404110; doi:10.1371/journal.pmed.1001270)

Figure S1. Funnel plot for studies meeting inclusion criteria providing an estimate for all CD4 counts. The Egger and Begg's p-values for funnel plot asymmetry are 0.02 and 0.12, respectively.

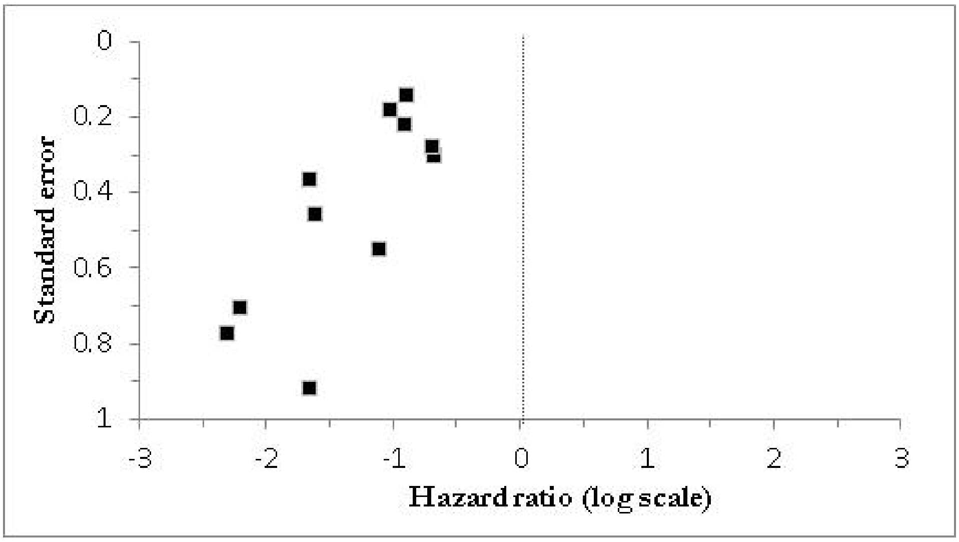

Supplement: Figure S1 — Funnel plot for studies meeting inclusion criteria, providing an estimate for all CD4 counts. (PDF) [file pmed.1001270.s001.pdf]
