# Supplementary material for: Antiretroviral Therapy for Prevention of Tuberculosis in Adults with HIV: A Systematic Review and Meta-Analysis
Source: PLoS Med. 2012 Jul 24;9(7):e1001270. doi: 10.1371/journal.pmed.1001270 (PMC3404110; doi:10.1371/journal.pmed.1001270)
Supplement: Table S1 — Search strategies for the PubMed, Embase, LILACS, and African Index Medicus databases. (PDF) [file pmed.1001270.s002.pdf]

Table S1. Search strategies for the PubMed, Embase, LiLACS, and AIM databases. The same search strategy was used for all databases.

Search number	Search terms
1	antiretroviral
2	anti-retroviral
3	HAART
4	cART
5	ART
6	ARV
7	1 or 2 or 3 or 4 or 5 or 6
8	tuberculosis
9	TB
10	8 or 9
11	risk*
12	rate*
13	hazard*
14	incidence*
15	odds*
16	11 or 12 or 13 or 14 or 15
17	7 and 10 and 16
