# Supplementary material for: Antiretroviral Therapy for Prevention of Tuberculosis in Adults with HIV: A Systematic Review and Meta-Analysis
Source: PLoS Med. 2012 Jul 24;9(7):e1001270. doi: 10.1371/journal.pmed.1001270 (PMC3404110; doi:10.1371/journal.pmed.1001270)
Supplement: Table S2 — HIV-associated tuberculosis cases in developed countries [3],[28]. (PDF) [file pmed.1001270.s003.pdf]

Table S2. HIV-associated tuberculosis cases in developed countries [3, 27]. – indicates no estimate

Country	HIV-TB cases	Human Development Index
Norway	< 10	0.938
Australia	52	0.937
New Zealand	< 10	0.907
United States	1100	0.902
Ireland	16	0.895
Liechtenstein	-	0.891
Netherlands	52	0.890
Canada	140	0.888
Germany	97	0.885
Sweden	16	0.885
Japan	100	0.884
Korea, Republic of	330	0.877
Switzerland	36	0.874
France	360	0.872
Israel	18	0.872
Finland	< 10	0.871
Iceland	< 10	0.869
Belgium	55	0.867
Denmark	15	0.866
Spain	950	0.863
Hong Kong, China (SAR)	42	0.862
Greece	12	0.855
Italy	180	0.854
Luxembourg	< 10	0.852
Austria	21	0.851
United Kingdom	310	0.849
Singapore	81	0.846
Czech Republic	< 10	0.841
Slovenia	-	0.828
Andorra	-	0.824
Slovakia	< 10	0.818
Malta	< 10	0.815
United Arab Emirates	< 10	0.815
Estonia	350	0.812
Cyprus	61	0.810
Brunei Darussalam	< 10	0.805
Hungary	15	0.805
Qatar	-	0.803
Bahrain	11	0.801
Poland	190	0.795
Portugal	690	0.795
Barbados	< 10	0.788
**Total reported in developed countries**	5410	
**Global**	1100000	
