# Supplementary material for: Antiretroviral Therapy for Prevention of Tuberculosis in Adults with HIV: A Systematic Review and Meta-Analysis
Source: PLoS Med. 2012 Jul 24;9(7):e1001270. doi: 10.1371/journal.pmed.1001270 (PMC3404110; doi:10.1371/journal.pmed.1001270)
Supplement: Text S2 — Calculation of incidence rate ratios and 95% confidence intervals. (PDF) [file pmed.1001270.s005.pdf]

Text S2. Calculation of incidence rate ratios and 95% confidence intervals

Reference for all calculations: Rothman KJ, Greenland S. Modern epidemiology. 3rd ed. Philadelphia: Lippincott Williams & Wilkins, 2008.

Cohen MS, Chen YQ, McCauley M, Gamble T, Hosseinipour MC, et al. (2011) Prevention of HIV-1 Infection with Early Antiretroviral Therapy. N Engl J Med 365: 493-505.

An effect estimate and 95% confidence interval was not reported in this study. However, the data necessary to calculate a rate ratio has been provided in the supplementary appendix. First, we must calculate the point estimate:

$$\widehat{IR} = \frac{A_1/T_1}{A_0/T_0}$$

A1 = 17 = tuberculosis cases in immediate ART arm
T1 = 1661.9 = person-years at risk of a clinical event in immediate ART arm

A0 = 33 = tuberculosis cases in deferred ART arm
T0 = 1641.8 = person-time at risk of a clinical event in deferred ART arm

IR = (17/1661.9) / (33/1641.8) = 0.5089

Next, we must calculate the standard deviation of the log rate ratio:

$$\widehat{SD}[\ln(\widehat{IR})] = \left(\frac{1}{A_1} + \frac{1}{A_0}\right)^{1/2}$$

SD[ln(IR)] = (1/17 + 1/33)^0.5 = 0.2985

Finally, we can calculate the lower and upper limits of the rate ratio:

$$\underline{IR}, \overline{IR} = \exp\{\ln(\widehat{IR}) \pm Z_\gamma \widehat{SD}[\ln(\widehat{IR})]\}$$

=exp[ln(0.5089) ± 1.96(0.2985)] = 0.2835, 0.9136

Therefore the rate ratio and its 95% confidence interval is: 0.51 (0.28 to 0.91).

Lannoy LH, Cortez-Escalante JJ, Evangelista Mdo S, Romero GA (2008) Tuberculosis incidence and risk factors among patients living with HIV/AIDS in public health service institutions in Brasilia, Federal District. Rev Soc Bras Med Trop 41: 549-555.

An effect estimate and 95% confidence interval was not reported in participants with baseline CD4 counts < 200 cells/µL. However, the data necessary to calculate a rate ratio has been provided. In order to calculate the tuberculosis rate ratio for people with CD4 counts < 200 cells/µL we must use the incidence rates and their 95% confidence intervals.

The incidence rate in people who started ART with baseline CD4 counts < 200 cells/µL was 0.60 cases / 100 person-years of observation (95% CI, 0.15 to 2.37). Given

$$\widehat{SD}[\ln(\widehat{IR})] = \frac{1}{A^{1/2}}$$

we can solve for the (1/A^0.5), i.e. the standard deviation of the log incidence rate, using the lower 95% confidence interval:

$$\underline{IR}, \overline{IR} = \exp[\ln(\widehat{IR}) \pm Z_{\gamma}(1/A^{1/2})]$$

ln(95% IR Lower Limit, LL) = ln(IR) – Zy SD[ln(IR)]

(ln(LL) - ln(IR)) / -Zy = SD[ln(IR)]

SD[ln(IR)] = (ln(0.15)–ln(0.60)) / -1.96 = 0.7073

Given SD[ln(IR)] we can now calculate A1, or the number of events in the stratum on ART:

$$\widehat{SD}[\ln(\widehat{IR})] = \frac{1}{A^{1/2}}$$

0.7073 = 1/(A1^0.5)
1 = 0.7073*(A1^0.5)
1 / 0.7073 = (A1^0.5)
1.4138 = (A1^0.5)
1.9989 = A1
A1~2 cases of tuberculosis

The incidence rate in people off ART with baseline CD4 counts < 200 cells/μL was 5.47 cases / 100 person-years of observation (95% CI, 2.73 to 10.94). Given

$$\widehat{SD}[\ln(\widehat{IR})] = \frac{1}{A^{1/2}}$$

we can solve for the (1/A^0.5), i.e. the standard deviation of the log incidence rate, using the lower 95% confidence interval:

$$\underline{IR}, \overline{IR} = \exp[\ln(\widehat{IR}) \pm Z_{\gamma}(1/A^{1/2})]$$

ln(95% IR Lower Limit, LL) = ln(IR) – Zy SD[ln(IR)]

(ln(LL) - ln(IR)) / -Zy = SD[ln(IR)]

SD[ln(IR)] = (ln(2.73)–ln(5.47)) / -1.96 = 0.3546

Given SD[ln(IR)] we can now calculate A0, or the number of events in the stratum off ART:

$$\widehat{SD}[\ln(\widehat{IR})] = \frac{1}{A^{1/2}}$$

0.3546 = 1/(A0^0.5)
1 = 0.3546(A0^0.5)
1 / 0.3546 = (A0^0.5)
2.8202 = (A0^0.5)
7.9537 = A0
A0 ~ 8 cases of tuberculosis

Since we have calculated the number of cases in both study arms, we can now calculate the rate ratio and its 95% confidence interval. First, we must calculate the point estimate:

$$\widehat{IR} = \frac{A_1/T_1}{A_0/T_0}$$

IR = (0.60/100) / (5.47/100) = 0.1097

Next we must calculate the standard deviation of the log rate ratio:

$$\widehat{SD}[\ln(\widehat{IR})] = \left(\frac{1}{A_1} + \frac{1}{A_0}\right)^{1/2}$$

SD[ln(IR)] = (1/2 + 1/8)^0.5 = 0.7912

Finally, we can calculate the 95% limits of the rate ratio:

$$\underline{IR}, \overline{IR} = \exp\{\ln(\widehat{IR}) \pm Z_{\gamma}\widehat{SD}[\ln(\widehat{IR})]\}$$

=exp[ln(0.1097) ± 1.96(0.7912)] = 0.0233, 0.5172

Therefore the rate ratio and its 95% confidence interval is: 0.11 (0.02 to 0.52).
